# CD133 expression correlates with clinicopathologic features and poor prognosis of colorectal cancer patients: An updated meta-analysis of 37 studies

**DOI:** 10.1097/MD.0000000000010446

**Published:** 2018-06-18

**Authors:** Rongyong Huang, Dan Mo, Junrong Wu, Huaying Ai, Yiping Lu

**Affiliations:** a School of Marine Sciences, Guangxi University; b Department of Surgery, Maternal and Child Health Hospital of The Guangxi Zhuang Autonomous Region, Nanning, Guangxi; c Department of Injection Room, The People's Hospital of Yingtan City, Yingtan, Jiangxi; d Department of Clinical Laboratory, The Affiliated Tumor Hospital of Guangxi Medical University, Nanning, Guangxi, China.

**Keywords:** CD133, colorectal cancer, prognosis

## Abstract

**Background:**

CD133 has been identified as a putative cancer stem cell marker in colorectal cancer (CRC). However, the clinicopathological and prognostic significance of CD133 in CRC patients remains controversial. Thus, we conducted a meta-analysis to quantitatively evaluate the above issues.

**Methods:**

We collected a comprehensive literature search from PubMed, Web of Science, and Embase database up to September 20, 2016 examining CD133 and clinical features of colorectal cancer patients. We used the odds ratio (OR) with 95% confidence interval (CI) to estimate the effects by overall and stratified analysis.

**Results:**

The overall result of our meta-analysis indicated that CD133 expression was positively correlated with T category, distant metastasis, lymphatic invasion, and vascular invasion. Moreover, patients with higher CD133 expression had a poorer overall survival (OS) (HR=2.01, *P < *.001) and a lower 5-year OS rate (OR = 3.26, *P < *.001) than those with lower expression. Disease-free survival (DFS) and 5-year DFS rate were similar with the above results. Though the correlation between CD133 expression with the clinical characteristic was not positive in some ways when we analyzed the different subgroup. The prognostic value of CD133 expression for 5-year OS rate of CRC patients was noticeable in spite of different patients’ region, multiple antibodies used in studies, various cut-off values of CD133 expression, and adjuvant therapy situation of patients.

**Conclusion:**

CD133 is a useful predictive or prognostic biomarker for CRC in clinical assessment and may serve as a potential therapeutic target for CRC.

## 1 Introduction

Colorectal cancer (CRC) is the third most common cancer and the fourth most common cause of cancer-related death worldwide.^[1]^ Although its diagnosis and therapy have been improved gradually, the survival of patients with CRC remains poor, which are mainly affected by drug resistance, local recurrence, and development of metastatic disease.^[2]^ Increasing research studies have shown that cancer stem cells (CSCs) with the principal properties of multipotency and self-renewal may be responsible for neoplasm formation, metastasis, recurrence, and therapeutic resistance.^[3–5]^

Cancer stem cells were successfully isolated and identified in many hematologic and solid tumors including colorectal cancer.^[5]^ A variety of molecules have been investigated as putative markers of CSCs in CRC. Among the various markers, CD133 is one of the most robust surface marker of CSCs in CRC.^[6]^ It is widely expressed in numerous types of solid tumors, involving CRC.^[7]^ As a 5 transmembrane single-chain glycoprotein, CD133, with a molecular weight of 120 kDa, was first found to be expressed in hematopoietic stem and progenitor cells by Yin et al.^[8]^ Also the exploration of CD133 as a surface marker of colon cancer stem cells is still in progress. In 2007, O’Brien et al^[9]^ found that CD133^**+**^ cells in CRC had the ability to initiate tumor growth.

The paradigm of CD133 as a CSCs biomarker has stimulated numerous studies to explore the prognostic power of CD133 expression in CRC patients. However, the prognostic value of CD133 for colorectal cancer remains controversial despite of numerous independent studies. For example, Kashihara et al and other studies demonstrated that high CD133 expression in CRC correlated with poor clinical outcomes.^[10–13]^ Nevertheless, Hong et al and other studies showed that CD133-negative patients exhibited a poor prognosis.^[14,15]^ Therefore, we performed a meta-analysis to elucidate the correlation between CD133 and clinicopathological features of CRC, and determine the value of CD133 as a prognostic marker for CRC.

## 2 Materials and methods

### 2.1 Literature search strategy

We collected a comprehensive literature search from PubMed, Web of Science, and Embase database up to March 20, 2016. The following search terms were used, (“colorectal cancer” or “rectal cancer” or “colon cancer”) and (“CD133” or “prominin 1” or “ac133 antigen” or “AC133 antigen”).

### 2.2 Study selection criteria

Eligible studies were included when the following criteria were met: the expression of CD133 protein on the cancer tissue (via either surgical or biopsy) by immunohistochemistry, rather than serum or any other kinds of detection methods was investigated; the association between CD133 and clinicopathological characteristics or the association of CD133 overexpression on disease free survival (DFS) and overall survival (OS) of CRC was studied; sufficient published data for calculating an odds ratio (OR), hazard ratio (HR), and 95% confidence interval (CI) were reported; (4) a full-text article in English or Chinese was published. When there were multiple articles by the same group based on similar patients and using the same detection methods, only the most important article with the recent or most information was included into the meta-analysis.

### 2.3 Data extraction

A standard protocol was applied to extract data. For every eligible study, the following data were sought: the first author's name, publication year, original country, situation of patient (whether received the adjuvant therapy, such as chemotherapy or radiotherapy before undergoing surgery), dilution of the used antibody, the choice of cut-off scores for the definition of positive staining, and survival analysis. We mainly clarified the association between CD133 expression and clinicopathological parameters, including T category, N category, distant metastasis, histology, lymphatic invasion, vascular invasion, and tumor size. More importantly, we investigated the association between CD133 expression and OS/ DFS. HR was directly extracted and synthesized from multivariable analysis where available. For those articles that only plotted as Kaplan–Meier curve, the software GetData Graph Digitizer 2.24 (http://getdata-graph-digitizer.com/) was used to digitize and extract the data of the 5-year OS rate and 5-year DFS rate directly. This investigation was approved by the institutional ethics committee, the Affiliated Tumor Hospital of Guangxi Medical University.

### 2.4 Statistical analysis

The strength of the association between the CD133 expression and clinicopathological features or 5-year OS/DFS rate were assessed by OR with the corresponding 95%CI. Moreover, the estimated HR with the corresponding 95%CI was used to summarize the relationship between CD133 expression and OS/DFS. In the present study, an OR>1 indicated a higher probability of tumor progression and poorer prognosis in CRC with CD133 overexpression, meanwhile a combined HR>1 implies a worse prognosis in the group with high CD133 expression. In the course of data pooling, statistical heterogeneity was performed by using chi-square-based *Q*-test. The *I*^2^ value indicates the degree of heterogeneity. A *P*-value < .10 and/or *I*^2^>50% are considered significant heterogeneity, and then a random-effect model is used.^[16]^ Otherwise, a fixed-effect model is used.^[17]^ Moreover, stratified-analyses were conducted based on patients’ district, antibodies used in studies, cut-off values of CD133 expression and adjuvant therapy situation of patients to explore the potential source of heterogeneity. Sensitivity analyses, by which each study was omitted in each turn to confirm the influence of individual data set to the pooled OR, were implied to evaluate the robustness of the results. Furthermore, we estimated potential publication bias with funnel plot and Egger's linear regression test. The funnel plot is visual symmetrical and the *P*-value of Egger's test is >0.05, which indicate that there is no statistically significant publication bias. All statistical tests in the meta-analysis were two-tailed and *P*-value ≤ .05 was considered statistically significant. Statistical analyses were performed with STATA software version 12.0.

## 3 Results

### 3.1 Characteristics of eligible literature

Based on the selection criteria, a total of 37 studies published from 2008 to 2016 were eligible for the meta-analysis.^[2,10–15,18–47]^ A total of 5397 CRC patients from China, Japan, South Korea, Italy, Germany, Australia, Spain, and Czech were enrolled. The study sample sizes ranged from 32 to 523 cases. All specimens were derived from CRC tissues by either biopsy or surgical resection, and were detected by immunohistochemistry (IHC) method. However, besides the antibody source and the dilution ratio, it is worth noting that there was no universally accepted standard about the cut-off value of the low expression or overexpression for CD133.

**Figure 1:**
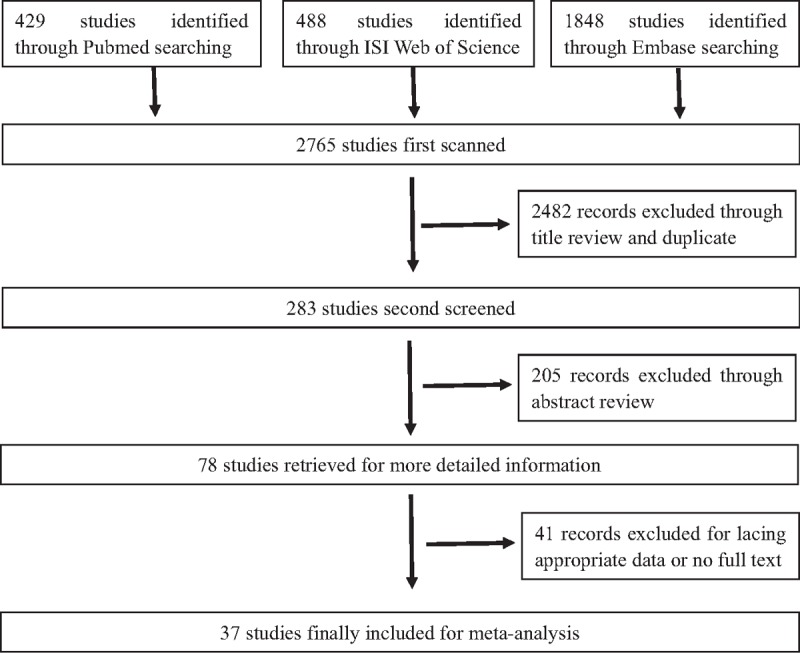
The flowchart of the study selection process.

Around 27 clinical studies assessed CD133 expression and correlated it to tumor clinicopathological characteristics which included tumor size, T category, N category, histological grade, distant metastasis, lymphatic invasion, and vascular invasion. Furthermore, 27 articles were analyzed for the association between CD133 expression and prognosis, which included 5-year OS, 5-year DFS, HR of OS, and HR of DFS. The flow diagram of study selection procedure was shown in Figure 1 and the main characters of the 37 eligible studies were summarized in Table 1.

**Table 1:**
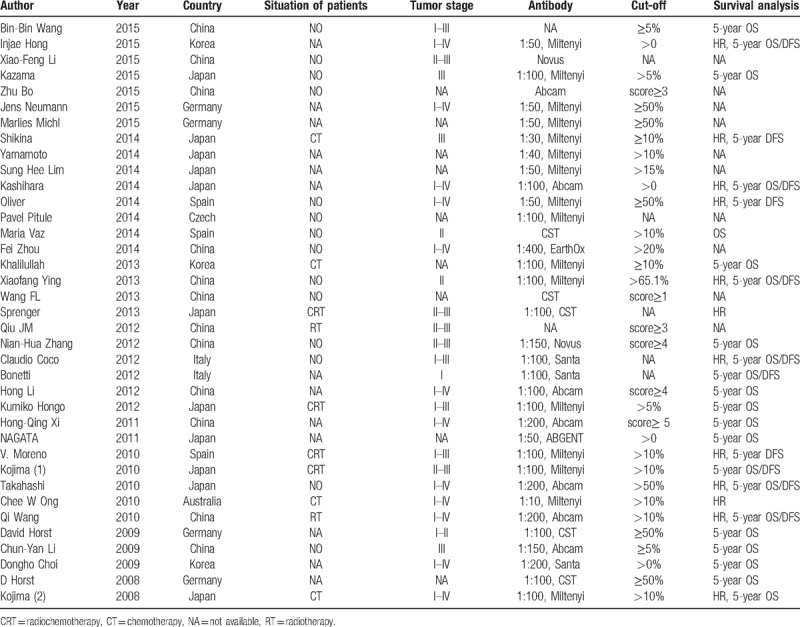
Main characteristics of the eligible studies.

### 3.2 Meta-analysis results

#### 3.2.1 Correlation of CD133 expression with clinicopathological characteristics

**Figure 2:**
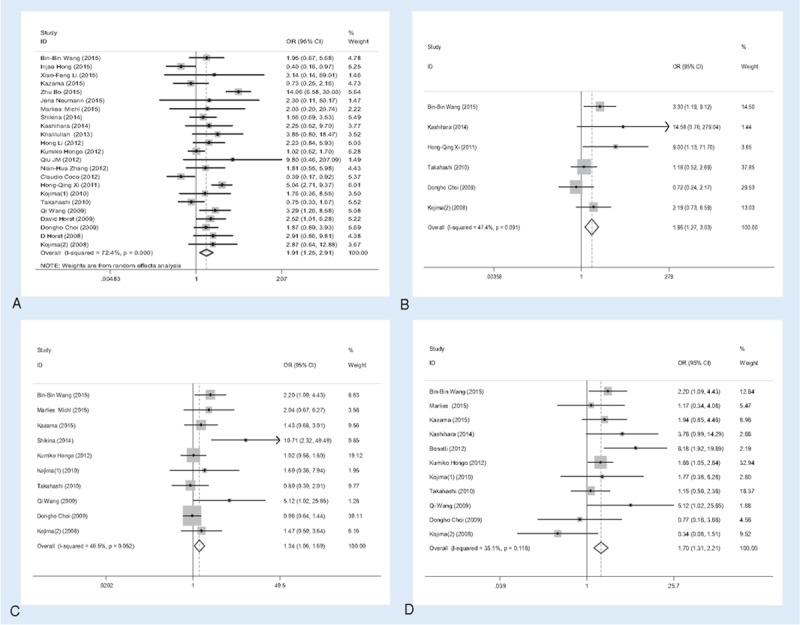
Association of CD133 expression with clinicopathological parameters. High CD133 expression was significantly associated with tumor T category (A), distant metastasis (B), lymphatic invasion (C), and vascular invasion (D).

**Overall analysis.** There were 27 studies that investigated the association between CD133 expression and tumor clinicopathological parameters. The overall analysis showed CD133 expression statistically significant correlated with T category (OR = 1.91, 95%CI = 1.25–2.91, *P = *.003, Fig. 2A), distant metastasis (OR = 1.96, 95%CI = 1.27–3.03, *P = *.002, Fig. 2B), lymphatic invasion (OR = 1.34, 95%CI = 1.06–1.69, *P = *.014, Fig. 2C) and vascular invasion (OR = 1.78, 95%CI = 1.22–2.59, *P < *.001, Fig. 2D). Specifically, higher CD133 expression means higher T category (T3+T4), greater possibility of distant metastasis, lymphatic invasion, and vascular invasion. However, no clear correlation was found between CD133 expression and histological grade (OR = 0.87, 95%CI = 0.60–1.27, *P = *.479), N category (OR = 1.18, 95%CI = 0.76–1.83, *P = *.464), tumor size (OR = 1.10, 95%CI = 0.82–1.49, *P = *.526), age (OR = 0.78, 95%CI = 0.48–1.27, *P = *.327), and sex (OR = 1.07, 95%CI = 0.93–1.24, *P = *.344). The results were summarized in Table 2.

**Table 2:**
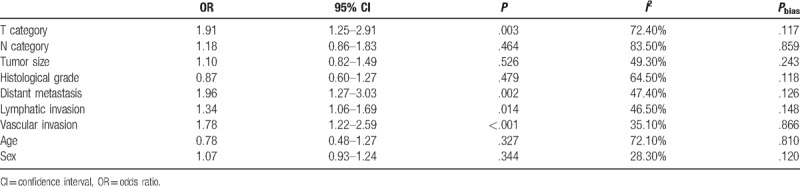
Overall analysis of CD133 expression association with clinical features.

**Subcategory analysis.** Because there were large heterogeneity in some groups, we explored the potential source of heterogeneity via stratified analysis based on patients’ district (America–Europe, Asia subgroups), antibodies used in studies (Miltenyi Biotec, Abcam, and others subgroups), cut-off values of CD133 expression (>50%, >5%/10% and others subgroups), adjuvant therapy situation of patients (With, Without, and NA subgroups). We discussed the main clinicopathological parameters in the followed subgroup analysis, which included T category, N category, histological grade, lymphatic invasion, and vascular invasion. It is worth mentioning that the degree of heterogeneity was apparently reduced by the stratified analysis. The results were summarized in Table 3.

**Table 3:**
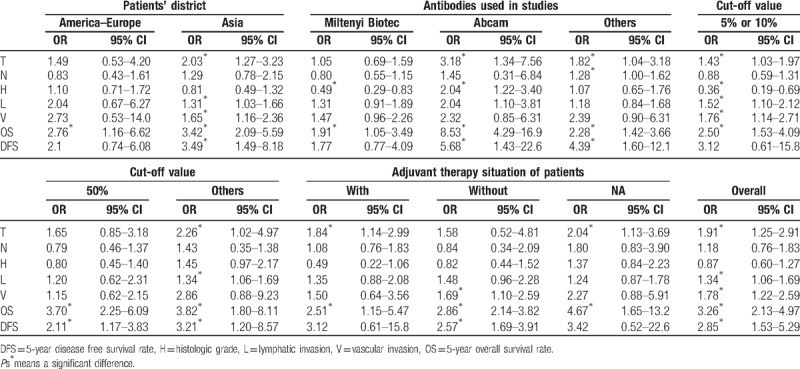
Main stratified analysis results of meta-analysis of CD133 expression.

In the subgroup analysis about the patients’ district, we found that CD133 expression was positively correlated with T category, lymphatic invasion and vascular invasion in the Asia subgroup, which was consistent with the results derived from the overall analysis. However, no above clinicopathological parameters were positively correlated with CD133 expression in the America–Europe subgroup. Besides that, N category and Histologic type was not correlated with CD133 expression regardless patients’ district.

For subcategory analysis about the antibodies, results were different among various subgroups. T category and histologic grade were positively correlated with CD133 expression in the Abcam group. However, in the Miltenyi Biotec group, CD133 expression was negatively correlated with histological grade and not correlated with the tumor T category and lymphatic invasion, which was not consistent with overall analysis. In the other antibody group, only T category and N category were positively related with CD133 expression.

In the subgroup analysis based on the cut-off value, for the subgroup studies with cut-off values >5%/10% and other subgroup, the results were nearly consistent with the results derived from overall analysis. In the cut-off values >50% subgroup, we found that there were no relationship between CD133 expression and clinicopathological parameters. N category was not correlated with CD133 expression regardless of cut-off value.

In the stratified analyses by the adjuvant therapy situation of patients, CD133 expression was positively correlated with the T category With adjuvant therapy situation of patients and NA subgroup, which was in accord with overall analysis. In the Without adjuvant therapy situation of patients’ subgroup, only vascular invasion was related to CD133 expression.

#### 3.2.2 Impact of CD133 expression on survival for colorectal cancer patients

The meta-analysis was performed on 27 studies investigating the association of CD133 expression and prognosis of CRC patients. The prognosis was evaluated by the indicators included 5-year OS, 5-year DFS, HR of OS, and DFS.

**Figure 3:**
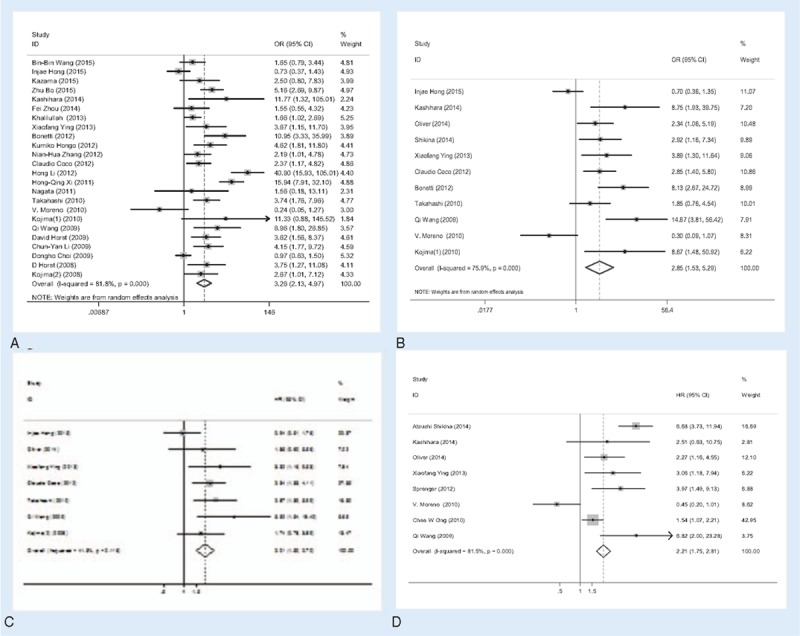
Meta-analysis of the association between CD133 expression and prognosis indicator. (A) 5-year OS; (B) 5-year DFS; (C) OS; (D) DFS. DFS = 5-year disease free survival rate, OS = 5-year overall survival rate.

**Overall analysis.** The data for this analysis indicated that the prognosis of colonrectal cancer patients with CD133^+^ was poorer than that of the CD133^−^ patients regardless of the indicators used. CD133 expression was highly correlated with low 5-year OS rate (OR = 3.26, 95% CI = 2.13–4.97, *P < *.001) and low 5-year DFS rate (OR = 2.85, 95% CI = 1.53–5.29, *P = *.001). Furthermore, the patients with higher CD133 expression presented poorer OS and DFS, and the pooled HRs were significant at 2.01 (95%CI = 1.50–2.70, *P < *.001) and 2.53 (95%CI = 1.36–4.70, *P = *.003), respectively. This indicated that CD133 was an independent prognostic factor in colon cancer patients. The results were shown in Figure 3 and Table 4.

**Table 4:**
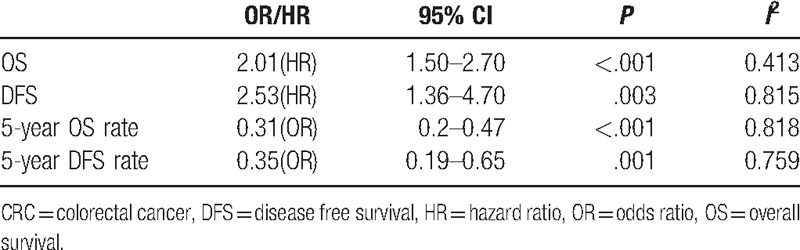
Overall analysis of CD133 expression association with prognosis indicator of CRC.

**Subcategory analysis.** On account of large heterogeneity in the 5-year OS rate (*P*_H_ < .001, *I*^2^ = 81.8%) and 5-year DFS rate (*P*_H_ = .001, *I*^2^ = 75.9%) analysis, we performed a subcategory analysis as described above. Higher CD133 expression was related to poorer 5-year OS regardless of subgroup. In the subgroup analysis about the patients’ district, the pooled OR (OR = 3.42, 95%CI = 2.09–5.59, *P < *.001) in the Asia district was higher than the pooled OR (OR = 2.76, 95%CI = 1.16–6.62, *P = *.022) in the America–Europe district. Meanwhile, the 5-year OS in the Abcam (OR = 8.53, 95%CI = 4.29–16.95, *P < *.001) was higher than that in the Miltenyi Biotec (OR = 1.91, 95%CI = 1.05–3.49, *P = *.034), and in others (OR = 2.28, 95%CI = 1.42–3.66, *P = *.001) antibody group. In the cut-off values subgroup, we found that the 5-year OS in the others (OR = 3.82, 95%CI = 1.80–8.11, *P < *.001) was higher than that in the >50% and the 5% or 10% group. Besides, the pooled OR with (OR = 2.51, 95%CI = 1.15–5.47, *P = *.024) was lower than that in the NA and Without adjuvant therapy group. However, higher CD133 expression related to poorer 5-year DFS rate was not found in the America–Europe, Miltenyi Biotec, cut-off >5%/10%, with and NA subgroups.

#### 3.2.3 Sensitivity analysis and Publication bias

**Figure 4:**
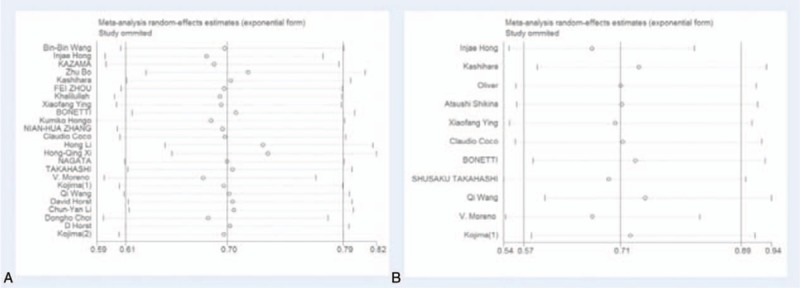
The plot of sensitivity analysis for evaluating the association between CD133 expression and 5-year OS/DFS. (A) 5-year OS; (B) 5-year DFS. DFS = 5-year disease free survival rate, OS = 5-year overall survival rate.

**Figure 5:**
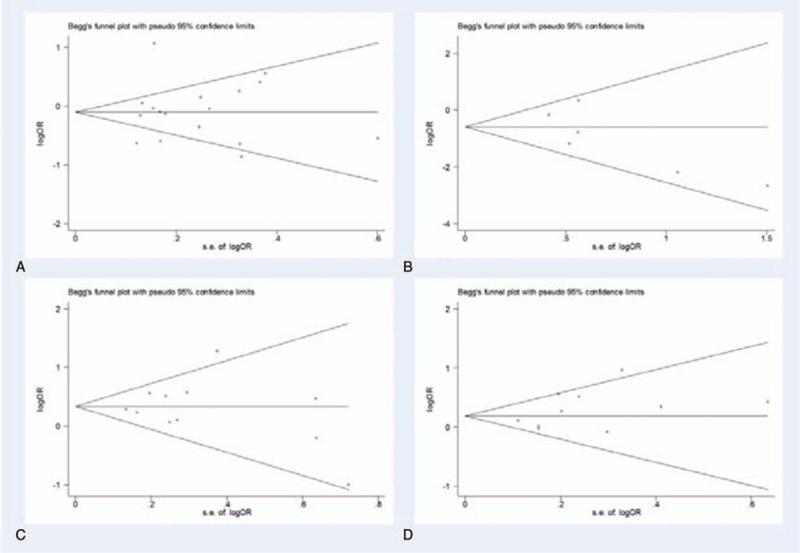
Begg's funnel plot of publication bias for evaluating the CD133 expression association with clinicopathological parameters of CRC. (A) T category; (B) distant metastasis; (C) lymphatic invasion; (D) vascular invasion. CRC = colorectal cancer.

Sensitivity analysis was performed through the sequential omission of individual studies. Moreover, no single study could essentially change the results, demonstrating that the results of our meta-analysis were statistically stable. The shapes of funnel plots and Egger's linear regression test were used to evaluate the publication bias of the eligible literatures. In general, the funnel plots did not show obvious evidence of asymmetry and the *P*-value of Egger's test was > 0.05, indicating there was no publication bias in the meta-analysis of CD133 and clinicopathological features (Figs. 4 and 5). The detailed results for *P*-value of Egger's test were summarized in Table 2.

## 4 Discussion

Lately, some researches have shown that CD133 may be used as a marker for colorectal CSCs.^[48]^ Many researchers have studied the relationship of CD133 expression with clinical features as well as prognostic indicator of CRC. However, there was disputed despite of abundant studies. Our meta-analysis results based on the existing 37 studies revealed that CD133 would be served as a poor predictive indicator in CRC patients. CD133 overexpression was positively associated with T category, distant metastasis, lymphatic invasion, and vascular invasion. Moreover, CD133 expression was correlated with lower 5-year OS/DFS rate and higher HR of OS/DFS in CRC patients.

In this meta-analysis, we firstly investigated the pooled association between CD133 expression and clinicopathological features. The high expression of CD133 was positively correlated with higher T category, distant metastasis, lymphatic invasion, and vascular invasion. Also this almost was in line with the report by Wang et al.^[12]^ Because there were large heterogeneity in some groups, we explored the potential source of heterogeneity via subcategory analysis based on the patients’ district, antibodies used in studies, different cut-off values of CD133 expression, and adjuvant therapy situation of patients. It was interesting to note that the result was different among different subgroups. In the Asian and cut-off >5/10% subgroup, what we found was almost consistent with the results derived from the overall analysis. Nevertheless, in the America–Europe subgroup, there was no correlation between CD133 and clinicopathological features, which may be attributed to the differences in gene and environment among ethnicity. Regarding the Miltenyi Biotec and others subgroup, the results were different from the overall analysis, which may be on account of different dilution rate used in the Miltenyi Biotec subgroup and various antibodies used in the others subgroup. However, there was few correlation between CD133 expression and clinicopathological features in the cut-off >50% and others cut-off subgroup. It may be due to a small number of literatures in the cut-off >50% subgroup and diverse cut-off value used in the others subgroup. Based on the different adjuvant therapy situation of patients’ subcategory analysis, the results were not consistent with the overall analysis, which may be on account of large heterogeneity and no unified adjuvant therapy in these subgroups. In addition, there was no significant correlation between CD133 expression and the tumor size. The small number of included studies might be the reason for this result. The results of our meta-analysis may support the hypothesis that CD133 overexpression might contribute to malignant progression of CRC, which may subsequently leads to a poor prognosis.

We then assessed the relationship between CD133 expression and prognostic significance of CRC. The pooled data indicated that high CD133 expression significantly predicted worse OS, poorer DFS, lower 5-year OS, and 5-year DFS rate. Indeed, accumulating evidence indicated that the CD133 was involved in the initiation and progression of CRC and associated with poor clinical outcomes. By combining the HRs and 95%CIs from primary studies, we showed that elevated CD133 expression was associated with poor OS (HR = 2.01; 95%CI = 1.50–2.70, *P* < .001) and poor DFS (HR = 2.21; 95%CI = 1.75–2.81, *P < *.001) in CRC. Meanwhile, we analyzed the relationship between CD133 expression and 5-year OS/DFS. The pooled overall results demonstrated that high CD133 expression significantly predicted low 5-year OS and 5-year DFS of CRC patients. In the following subcategory analysis, the 5-year OS in any subgroup was in accordance with the overall analysis. However, the positive correlation between CD133 expression and the 5-year DFS could not be found in the America–Europe, Miltenyi Biotec, cut-off >5/10%, With, and NA subgroups. It may due to differences in gene and environment among ethnicity, different dilution rate used, diverse adjuvant therapy applied in the above subgroups. It is notable that there is association of CD133 expression with prognostic significance of CRC in our meta-analysis, suggesting that this marker can be developed for the prognostic assessment and clinical targeted therapy.

Several studies have shown that CSC-related factors, including aldehyde dehydrogenase 1 (ALDH1) and leucine-rich repeat containing G protein-coupled receptor 5 (LGR5), are associated with colorectal cancer progression.^[49–51]^ As for the prognostic value of these markers, Horst et al^[45]^ implied that CD133 might be of the most clinical relevance, while the combined evaluation of CD133, CD44, and CD166 might even be more valuable to separate high-risk groups from low-risk colorectal cancer cases. For future studies, co-expression of colorectal CSC markers associated with patient survival may be more serviceable for clinical application in CRC.

Although we executed exhaustive meta-analysis, certain potential limitations existed and some results needed to be elaborated deliberately. Firstly, we only directly extracted HRs and 95% CIs from the original study and we did not calculate HRs and 95% CIs in other ways, which may omit much important information. Secondly, we assessed the 5-year OS/DFS rate from the Kaplan–Meier curves. These estimated data may be less credible than direct data from the original study. Thirdly, in spite of modification for heterogeneity by the application of random-effect model, subcategory analyses and sensitivity analyses, there was large heterogeneity in some subgroups. Fourthly, though we evaluated the publication bias and did not discover obvious bias, it is a remarkable fact that the papers with positive results are prone to publishing. Therefore, the relevancy between CD133 expression and outcome of CRC patients may have exceeded our calculation. Furthermore, studies measuring CD133 gene or mRNA level by RT-PCR was not yet included in this meta-analysis. Finally, except for CD133, this present study did not examines the correlation between other putative CSC markers and the risk of CRC.

In summary, although certain limitations existed, the results of the present study showed that higher CD133 expression was positively correlated with higher T category, lymphatic invasion, and vascular invasion. Moreover, higher CD133 expression was associated with poorer prognosis of CRC. Our results suggest that CD133 expression not only provides a better understanding of the relationship between colon tumorigenesis but may also be a useful predictive or diagnostic biomarker for CRC and beneficial for the novel targeted therapeutic strategies in the future. However, a future larger prospective study may be needed to warrant to test our results.

## Author contributions

**Data curation:** Rongyong Huang, Huaying Ai, Yiping Lu.

**Formal analysis:** Rongyong Huang, Huaying Ai.

**Funding acquisition:** Junrong Wu.

**Investigation:** Rongyong Huang, Huaying Ai.

**Writing – original draft:** Rongyong Huang, Dan Mo.

**Writing – review & editing:** Rongyong Huang, Junrong Wu, Yiping Lu.
