# CD133 expression correlates with clinicopathologic features and poor prognosis of colorectal cancer patients: An updated meta-analysis of 37 studies: Erratum

**DOI:** 10.1097/MD.0000000000011956

**Published:** 2018-08-10

In the article, “CD133 expression correlates with clinicopathologic features and poor prognosis of colorectal cancer patients: An updated meta-analysis of 37 studies”,^[1]^ which appeared in Volume 97, Issue 23 of *Medicine*, the authors would like a change in author and affiliation order.

Yiping Lu, MD^1#^, Huaying Ai, MD^2#^, Dan Mo, MS^3^, Junrong Wu, MS^1^, Rongyong Huang, PhD^4∗^

^1^Department of Clinical Laboratory, The Affiliated Tumor Hospital of Guangxi Medical University, Nanning, Guangxi, China.

^2^Department of Injection Room, The People's Hospital of Yingtan City, Yingtan, Jiangxi, China.

^3^Department of Surgery, Maternal and Child Health Hospital of The Guangxi Zhuang Autonomous Region, Nanning, Guangxi, China.

^4^School of Marine Sciences, Guangxi University, Nanning, Guangxi, China.

^#^ Yiping Lu and Huaying Ai contributed equally to this work.
